# Assessment of Chestnut Gall Toughness: Implications for a Biocontrol Agent

**DOI:** 10.3390/insects13121095

**Published:** 2022-11-28

**Authors:** Chiara Ferracini, Cristina Pogolotti, Simone Giacosa, Eleonora Vittoria Fontana, Luca Rolle, Alberto Alma

**Affiliations:** Department of Agricultural, Forest and Food Sciences (DISAFA), University of Torino, Largo Paolo Braccini 2, 10095 Grugliasco, Italy

**Keywords:** *Torymus sinensis*, *Dryocosmus kuriphilus*, biocontrol agent performance, diapause, gall wasp, parasitoid-host interaction, mass rearing

## Abstract

**Simple Summary:**

*Torymus sinensis*, the biocontrol agent of the gall wasp *Dryocosmus kuriphilus*, is univoltine, and exhibits a prolonged diapause. Further investigations have been carried out to assess the extent of the diapause and its trend over the years. Moreover, the seasonal variation in the galls’ toughness was measured to assess if the wall of dry galls formed in the previous year was so hard to counteract *T. sinensis* emergence, thus negatively affecting diapause. The window of vulnerability of the galls was also evaluated in controlled conditions. The results showed that the average number of second year *T. sinensis* emerging per 100 cells was 0.41 ± 0.05, and dead adults accounted for 4.1 ± 0.23 per 100 cells. Gall toughness resulted in lower values for galls collected in May and June. In general, no difference was detected in the wall toughness of galls formed during the previous year when compared to current-year dry galls. Comparing the number of oviposition events by *T. sinensis* and the gall toughness, a negative correlation was found. Descriptive information on this gall’s structural traits and the influence on gall wasp management are also discussed.

**Abstract:**

(1) *Torymus sinensis*, the biocontrol agent of the Asian chestnut gall wasp *Dryocosmus kuriphilus*, is univoltine, but in NW Italy a small percentage of individuals exhibits a prolonged diapause, mainly as late instar larva. (2) In 2020, the diapause was investigated to evaluate its trend over the years. Due to the low survival rate of diapausing *T. sinensis* adults, the seasonal variation in the galls’ toughness was evaluated, thus assuming that dry galls over time can negatively affect emergence. The window of vulnerability of the gall wasp galls was also evaluated in controlled conditions. (3) The results showed that the average number of second year *T. sinensis* emerging per 100 cells was 0.41 ± 0.05, and dead adults accounted for 4.1 ± 0.23 per 100 cells. Gall toughness resulted in lower values for galls collected in May and June, and then gradually increased over time. In general, no difference was detected in the wall toughness of galls formed during the previous year when compared to current-year dry galls. Oviposition was recorded on all the tested galls collected in May and June, and no difference in the number of oviposition events was detected. Conversely, no oviposition was observed in July. Comparing the number of oviposition events by *T. sinensis* and the gall toughness, a negative correlation was found (R^2^ = −0.99). (4) The present findings contribute descriptive information on this gall’s structural traits, and the influence on gall wasp management is also discussed.

## 1. Introduction

Galls are pathologically developed cells, tissues or organs of plants that have arisen mostly by hypertrophy and hyperplasy under the influence of parasitic organisms, such as bacteria, fungi, nematodes, mites, or insects. They represent the growth reaction of plants to the attack of the parasite and are in some way related to the feeding activity and nutritional physiology of the parasite [1]. 

Many insect groups, and an estimated 13,000 species, induce plant galls [2]. Gall wasps (Hymenoptera: Cynipidae, Cynipini) constitute the second largest radiation of galling insects after gall midges (Diptera: Cecidomyiidae), and some of the most complex and well-organized galls are induced by gall wasps. Cynipid galls are found on all plant organs (i.e., flowers, leaves, buds, stems, twigs, and roots), and contain one to more than 100 larval chambers [3]. The most familiar cynipid gall inducers are associated with the Fagaceae (mainly *Quercus,* but also *Castanea*, *Chrysolepis* and *Lithocarpus*) and Rosaceae families, but there is also a significant number of herb-galling cynipids (Apiaceae, Asteraceae, Brassicaceae, Lamiaceae, Papaveraceae, Rosaceae, and Valerianaceae,) [3,4]. The oak gall wasps (Cynipini) are by far the most species-rich group of gall wasps, with about 1000 species in 25 genera worldwide [4].

The gall former alters the physiological state of plant tissues, particularly that of the cells nearest to the feeding larvae, the so-called nutritive tissue, which is maintained in a metabolically active state by the gall former [5]. Gall tissue is generally thought to be relatively high in nutrients and low in secondary compounds compared to ungalled plant tissue, even if discrepancies in the literature are also reported [5]. Galls are an integrated part of the plant, can alter plant architecture and reproduction, and often play active physiological roles, regulating lateral or adventitious regrowth after gall formation. They can reduce shoot growth, leaf area, and inflorescence development, but increase photosynthetic rates and stem water potential [6]. 

Cynipid gall development can be divided into three phases: initiation, growth, and maturation. Initiation begins with oviposition by the female gall wasp, determining host plant, gall location on the host, and the number of larvae developing in the resulting gall (in relation to the number of eggs laid) [7].

Structurally, galls induced by the sexual or asexual generation of gall wasps are divided into two larval chambers surrounded by an outer layer. Larval chambers are alike in most galls. Near each larval chamber there is a mass of nutritive cells surrounded with a single layer of parenchyma cells. In most galls, these two layers are covered with a third layer of sclerenchyma cells [8].

About 30 *Dryocosmus* species have been reported on *Castanea*, *Chrysolepis*, and *Quercus* in the world [4], the best known of which is the Asian chestnut gall wasp (ACGW) *Dryocosmus kuriphilus* Yasumatsu (Hymenoptera: Cynipidae). The ACGW, native to China, was first reported in Japan in 1941 [9] and introduced into Europe in 2002 with the movement of infested plant material [10]. It attacks the vegetative buds and disrupts shoot growth through the formation of galls [11]. The galls suppress shoot elongation and reduce fruiting, and severe infestations may result in the decline and death of chestnut trees [12,13]. The size of a gall is most likely directly related to many factors, such as wasp potential fecundity, which is positively correlated with gall size [14,15].

Galls are uni- or multilocular and contain from 1 to 25 larval chambers [14,16], localized on shoots, leaf midribs or leaf stipules [17]. After the emergence of *D. kuriphilus* adults, galls dry, become wood-like and remain on the tree for several years. ACGW galls are known to support species richness, closed communities of inquilines, and parasitoids that have become a model system in community ecology [7]. Soon after the introduction of *D. kuriphilus* in Italy, generalist native parasitoid species quickly recruited to this novel gall wasp host. Specifically, the community of native parasitoids recorded invading ACGW populations is mainly composed of chalcid species (Hymenoptera: Chalcidoidea), commonly known to be parasitoids of oak cynipid gall wasps. Although several families have been reported associated with the ACGW in its introduced range (e.g., Eulophidae, Eupelmidae, Eurytomidae, Ormyridae, Pteromalidae, Torymidae), they did not provide effective control of this pest [18,19,20].

To cope with this phytosanitary threat, the biocontrol agent *Torymus sinensis* Kamijo (Hymenoptera: Torymidae) was imported from Japan. The parasitoid was mass reared and released on a large scale in European chestnut-growing areas affected by the gall wasp [13,21]. This larval parasitoid performs one generation per year, but in the introduced new areas (NW Italy), it was found to exhibit a prolonged diapause mainly as late instar larva, showing a two-year life cycle. Due to the release of the parasitoid, the presence of the ACGW host has dramatically decreased by limiting the food availability for the population of this monophagous wasp. A prolongation of diapause was supposed to have an adaptive value in protecting the population against the yearly fluctuation in food supply [22]. Low diapause levels were recorded for *T. sinensis* (1–3%) [22,23].

Several papers have emphasized the importance of gall characteristics on the success of parasitoids when attacking gall insects, and size, thickness, toughness, and the parasitoid’s ovipositor length are considered to be important parameters affecting the parasitoid oviposition rate and success of gall-forming insects [24,25]. Hardening of the gall walls is considered one of the most important deterrents to further attack by parasitoids. In particular, tougher galls are thought to be harder to attack than softer galls because of the physical difficulty of drilling with an ovipositor, and thicker-walled gall may determine a lower rate of parasitoid attack [7,26]. 

The gall wall thickness is known to affect the ability of parasitoids to successfully attack the cynipids [8], and gall toughness might have a significant influence on gall success, as well. Previous investigations assessing gall thickness were performed using an ocular micrometer in the case of the gall-making fly *Eurosta solidaginis* (Fitch) (Diptera: Tephritidae) [27], while gall toughness was evaluated with a pressure dynamometer in the case of the galls of *Aditrochus coihuensis* Ovruski (Hymenoptera: Pteromalidae), but no information about the force of penetration was available [28]. The toughness of *Asphondylia fiocossa* Hawkins (Diptera: Cecidomyiidae) spring gall tissue was also measured using a similar texture analyzer approach [29], but even in this case the measurement error for gall toughness was high, and no data are available. 

In 2020, research was carried out to assess the extent of the diapause and if its rate had changed over the years, after the previous observations in 2015 [22]. The survey sites previously investigated were discarded due to the scarcity of galls due to the effective biological control programs carried out by releasing the biocontrol agent *T. sinensis* [20]. 

A prolonged dormancy may affect reproduction, exposing individuals to increased mortality, and both prolonged dormancy and increased mortality may result in fitness costs [22]. Mortality rates of newly formed second year *T. sinensis* adults (diapausing) inside the galls were already detected by gall dissection [22]. Thus, research was carried out to measure the toughness of ACGW galls, assuming that dry galls over time can harden to the point that adults cannot emerge. In particular, we investigated the following questions: (i) what is the seasonal variation in the ACGW galls’ toughness from formation to desiccation? (ii) is the wall of dry galls formed in the previous year so hard to counteract *T. sinensis* emergence, thus negatively affecting diapause? (iii) since galls can be located in different positions, is there any difference in toughness, comparing galls collected on branch vs. leaf midrib? 

Furthermore, we evaluated the window of vulnerability of the gall wasp galls (namely, a limited portion of the host life cycle susceptible to parasitism [24]), assessing if *T. sinensis* females are less inclined to lay eggs in tougher galls, limiting oviposition events to the period before galls mature and harden. To test the gall toughness hypothesis, we evaluated the suitability of fresh galls collected in different months (May, June and July) for *T. sinensis* oviposition, in controlled conditions.

## 2. Materials and Methods

### 2.1. Survey Sites

Investigations were performed in 2020–2021 in the municipality of Vicchio (43°57′44.7″ N 11°32′46.6″ E; 785 m a.s.l.), located in the Tuscany region (Northern Italy). The survey site was characterized by managed sweet chestnut orchards (*Castanea sativa* Miller var. Marrone del Mugello). Trees were approximately 80 yrs old, 20 m in height, planted at 10 m intervals along the row and with a 15 m distance between rows. Tree density was about 100 trees/ha. This survey site was chosen to ensure an adequate presence of galls: ACGW infestation index > 3, according to the index reported by Ferracini et al. [30]. 

### 2.2. Collection and Dissection of the Galls

To evaluate the extent of the diapause, in 2020, ten naturally growing chestnut trees were randomly chosen, and for each tree 500 galls were randomly collected (50 galls × 10 branches) on the crown of the plant during winter (January T_min_ = −2.9 °C, T_max_ = 11 °C, T_avg_ = 3.42 °C, RH_min_ = 25.7%, RH_max_ = 100%, RH_avg_ = 87.8%; February T_min_= −5.2 °C, T_max_= 24.2 °C, T_avg_ = 7.71 °C, RH_min_ = 15%, RH_max_ = 100%, RH_avg_ = 78.8%), and stored in rearing cardboard boxes in outdoor conditions [22]. The number of second-year adults emerging in spring 2022 was recorded, and the galls were then dissected. The dissection was conducted using a stereomicroscope with the aid of a scalpel. The parasitism rate and phenological stage of *T. sinensis* individuals (larvae, immature pupae, mature pupae, and newly formed adults, dead or alive) were evaluated.

To evaluate the seasonal variation in the galls’ toughness, in 2021, ten naturally growing chestnut trees were randomly chosen, and 60 galls of similar size (1.5 × 1.5 cm) were collected by the same operator each month, from early May to early December (no gall was already available in April in the survey site). Galls were identified as either branch galls (occurring on the chestnut shoot) or as leaf midrib galls (occurring along the leaf midrib), in order to have the same number of galls of both types (30 branch galls and 30 leaf midrib galls collected per month). Since gall morphology (volume and mass) may be influenced by exposure to sun and precipitation [31], collection was performed randomly on the crown of the plant, according to the methods described in Ferracini et al. [18]. Galls were excised, placed in plastic bags, kept in climatic bags, and stored in the fridge at 4 °C until toughness analysis (see specific section). Dissection was performed in laboratory conditions, according to the methods described in Ferracini et al. [22], and the parasitism rate and phenological stage of *T. sinensis* individuals were recorded.

Moreover, in 2021, ACGW fresh galls were collected to perform the oviposition trials (see specific section). A total of 100 fresh galls (10 galls × 10 plants) of similar size were collected in May, June, and July, for a total of 300 galls. To avoid any influence on the behavior of the parasitoid, chestnut galls were collected in a chestnut orchard characterized by a very low presence of the parasitoid. The collected galls were divided into two subsets. Half of the galls were used in the oviposition trials, and the remaining galls were dissected using a stereomicroscope to evaluate the parasitism rate by *T. sinensis*, which accounted for less than 20%, in accordance with previous investigations by Ferracini et al. [30].

### 2.3. Gall Toughness Analysis

120 dry galls (60 galls collected in the winter of 2019–2020 and 60 galls collected in the winter of 2020–2021), and 60 fresh galls (30 branch galls and 30 leaf midrib galls) collected each month from May to December were subjected to the evaluation of the instrumental mechanical properties within 24 h of field collection. A TA.XTplus texture analyzer, equipped with a HDP/90 platform and a P/2N needle probe (Stable Micro Systems, Godalming, Surrey, United Kingdom) was used. The load cell used was 50 kg, except for the material obtained during the 2021 season, which allowed the use of a 5 kg load cell to maximize load cell resolution [32]. 

**Figure 1:**
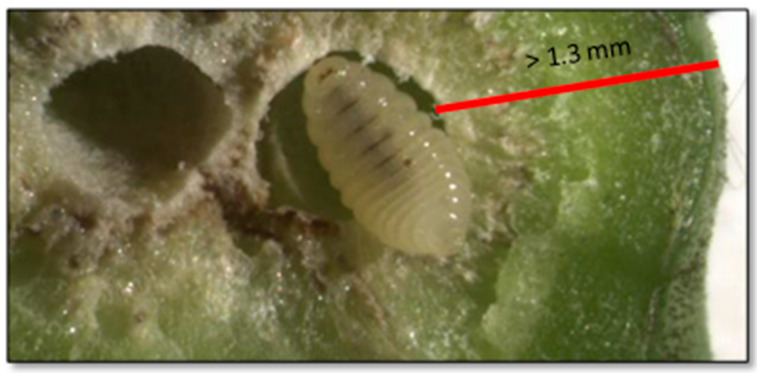
Example of the location of a larval chamber. The minimum depth was calculated in a representative sample of galls (N = 50) to set the depth of the needle in the deformation tests.

To evaluate the galls’ toughness, we conducted preliminary investigations to establish the depth of insertion of the needle probe, and to avoid the values being distorted by the presence of very superficial larval chambers. Specifically, a representative sample of galls (N = 50) of different types (dry and fresh branch and leaf midrib galls) and size (<1 × 1 cm, about 1 × 1 cm, >1.5 × 1.5 cm) was dissected under the microscope, highlighting how all the larval chambers were located at a depth of at least 1.3 mm (Figure 1). Thus, we decided to perform a 1-mm depth deformation test, repeated twice for each gall (on different positions chosen randomly). Considering the test distance selected and test operating conditions, a test speed of 0.2 mm/s was chosen after evaluation of test conditions derived from other studies [33], with the criteria of avoiding excessive stress on the material and maximizing the number of points acquired during each acquisition. 

**Figure 2:**
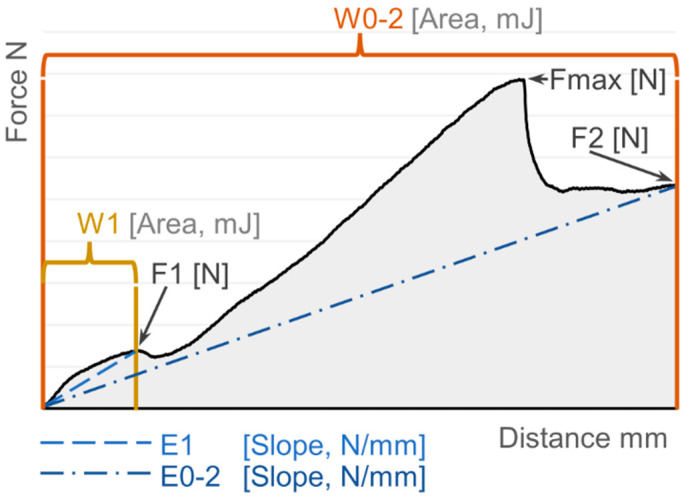
A typical distance-force curve obtained with the determination of instrumental mechanical properties (1-mm needle penetration). The calculated parameters are annotated on the graph.

For each test, the distance-force curve was acquired at 500 points per second, and the following parameters were determined (Figure 2): F1 (force opposed at the first force peak, Newton (N)), W1 (energy opposed between the initial test point and the first force peak, milliJoule (mJ)), E1 (force gradient between the initial test point and the first force peak, N/mm), F2 (force opposed at the end of the test, i.e., after 1 mm of deformation, N), W0-2 (energy applied during the whole test, mJ), E0-2 (force gradient between the initial and the final test point, N/mm), Fmax (maximum force applied during the whole test, N) (Figure 2).

### 2.4. Insects

*Torymus sinensis* adults were obtained from a mass rearing at the DISAFA laboratory. Mated six-day-old naïve females were used. One day before the trials, one female was placed in a plastic tube closed with a cotton plug, together with three males to ensure mating according to Ferracini et al. [34]. Individuals were provided drops of honey on cardboard and kept in a climatic chamber at 15 ± 1 °C, 60 ± 5% RH, and a photoperiod of 16:8 (L:D) h, until the trials.

### 2.5. Oviposition Trials

A single fresh *D. kuriphilus* gall was offered to a mated *T. sinensis* female placed on a filter paper sheet inside a Petri dish arena (diameter 10 cm) for 48 h, and 50 replications per month were performed. The number and duration of the oviposition behavioral event were recorded for 45 min using JWatcher^®^ 1.0 software (University of California, Los Angeles, CA, USA). Oviposition was considered successful when the female spent more than 60 s with the ovipositor inserted in the gall, according to Ferracini et al. [34]. Females were exposed to fresh galls collected in May, June and July. All the tested galls were individually stored in glass tubes (120 mm in height × 18 mm in diameter), and then dissected with the aid of a scalpel using a stereomicroscope. Since eggs may have escaped detection, galls were stored in a climatic chamber at 24 ± 2 °C, 50 ± 10% RH, and a photoperiod of 16:8 (L:D) h for ten days to ease the detection of the parasitoid at the larval stage. 

### 2.6. Statistical Analysis

The gall toughness results on dry and fresh galls, obtained using compression tests, were subjected to one-way analysis of variance (ANOVA), and significant differences were highlighted when *p* < 0.05. In this case, significant differences among samples were identified by performing a Tukey-HSD *post-hoc* test. 

In the behavioral trials, we used a linear regression to investigate the number of oviposition events by *T. sinensis*. After testing for homogeneity of variance (Levene’s test), data were analyzed using the Student’s *t* tests (*p* < 0.05) to compare the number of oviposition events occurring on galls collected in different months (May, June, and July) with different degrees of toughness. Moreover, the parasitism rate by *T. sinensis* for the galls collected in the three different months was assessed using a generalized linear model (GLM) following a binomial distribution (logit link function), comparing the number of *T. sinensis* larvae before and after the oviposition trials. The correlation between the number of oviposition events and gall toughness was investigated, as well. All statistical analyses were performed using R software (version 4.1.1; R Foundation for Statistical Computing, Vienna, Austria). The boxplot visualization was prepared with R software plus the ‘ggplot2’ package.

## 3. Results

### 3.1. Collection and Dissection of the Galls

In 2020, a total of 5000 galls was collected, and adult parasitoids emerged in the spring of the second year (2022), simultaneously with the emergence of univoltine adults observed in natural conditions. The average number of second year *T. sinensis* emerging per 100 cells was 0.41 ± 0.05, and dead adults accounted for 4.1 ± 0.23 per 100 cells (data on univoltine adults’ emergence are not shown).

### 3.2. Gall Toughness Analysis

**Figure 3:**
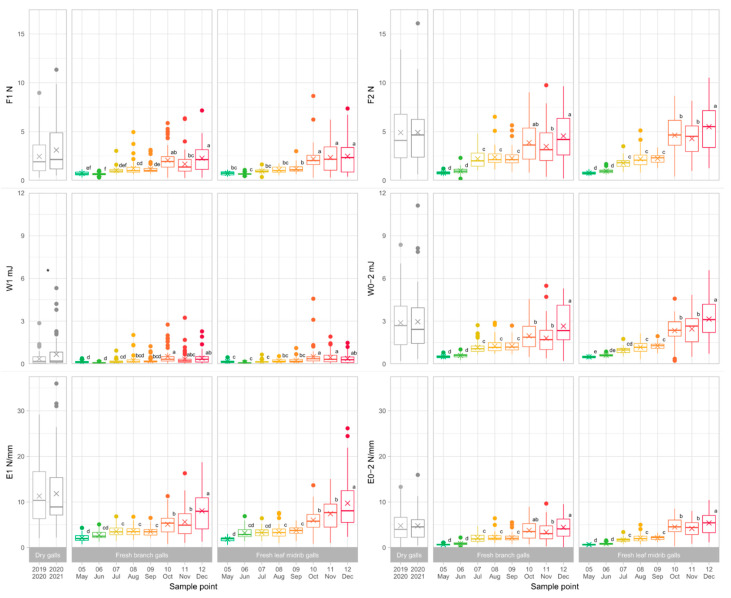
Evaluation of mechanical parameters on dry and fresh galls using a 1-mm needle compression test, distinguishing between fresh branch and leaf midrib galls (N = 60). The multiplication sign (×) represents the mean value for each sample. Outliers were represented with colored points. Inside each parameter and sample type (dry galls, fresh branch galls, fresh leaf midrib galls), different letters evidence significant difference at *p* < 0.05 (Tukey HSD *post-hoc* test).

**Figure 4:**
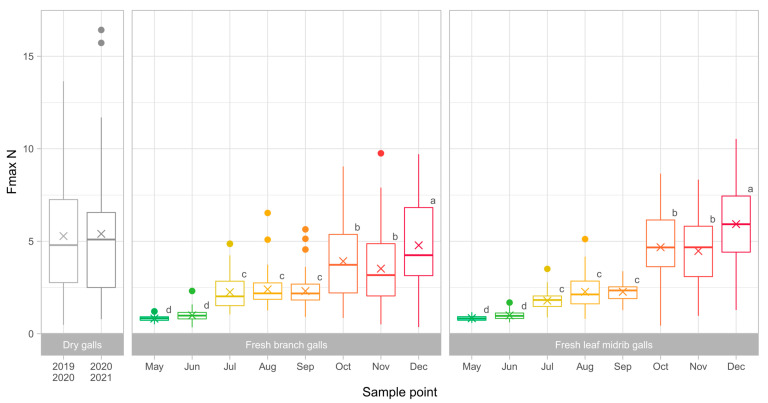
Maximum force (Fmax) recorded during the whole test, distinguishing between fresh branch and leaf midrib galls (N = 60). The multiplication sign (×) represents the mean value for each sample. Outliers were represented with colored points. Inside each sample type (dry galls, fresh branch galls, fresh leaf midrib galls), different letters evidence significant difference at *p* < 0.05 (Tukey HSD *post-hoc* test).

To assess the degree and trend of toughness of ACGW galls, 480 galls were collected in 2021 (240 branch galls and 240 leaf midrib galls). Figure 3 shows all the parameters recorded for branch and leaf midrib galls collected from May to December. The results showed that the gall toughness features, prominently, penetration force parameters, are lower in the case of galls collected in the first two months (0.66–0.74 N for F1, 0.82–0.99 N for Fmax; Figure 4), and then they gradually increase over time, with a more pronounced increase starting from July. All parameters, except W1, exhibited their peak for the galls collected in December (2.28–2.48 N for F1, 4.78–5.93 N for Fmax). This latter trait also increased over time, but its trend was less clear, with minimum values in June and a significantly increasing trend leading to October, when the maximum was achieved.

When comparing the two gall types analyzed, significant differences were found for all parameters except W1. F1 values among gall types were significantly different (*p* = 0.007) at the November sampling point, while E1 was able to significantly (*p* < 0.05) discriminate between the gall types collected in May, June, October, and November. F2, W0-2, E0-2, and Fmax parameters showed significant differences (*p* < 0.05) between the gall types considered at July, October, November, and December (excl. W0-2 for the latter) sampling points. Therefore, November was the sampling point in which the most gall toughness parameters were able to discriminate between the two gall types considered.

No significant difference was detected between dry winter galls collected in the current and previous year, except for the W1 deformation energy parameter (0.675 vs. 0.357 mJ, respectively; *p* < 0.05). A sustained variability of the results was found in these samples with respect to fresh branch and leaf midrib galls (Figure 3); however, the force parameter Fmax recorded at the end of the deformation (1 mm) showed similar (*p* > 0.05) results for both dry samples (5.28 and 5.40 N for material collected in 2019–2020 and 2020–2021 seasons, respectively) when compared with December branch and leaf midrib galls (4.78 and 5.93 N, respectively).

### 3.3. Oviposition Trials

**Figure 5:**
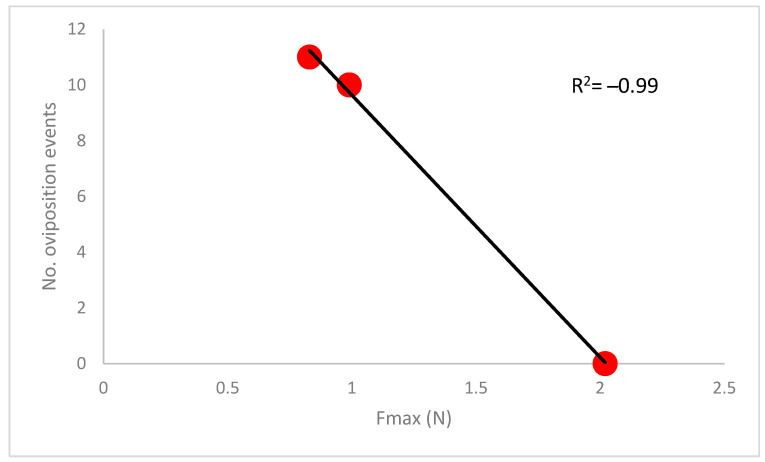
Number of oviposition events recorded in the oviposition trials (N = 50 galls per month) in relation to the toughness of the galls expressed as maximum force (Fmax).

Oviposition was recorded on all the tested galls collected in May and June, and no difference in the number of oviposition events was detected (*t*-test = 0.15; df = 98; *p* = 0.87). Specifically, during the 45′ observation period, 11 oviposition events were detected on galls collected in May and 10 in those collected in June, on average 20 and 16 min after the host location, respectively. Conversely, no oviposition was ever observed for galls collected in July, and each gall encountered was rejected. After 10 days of storage in the climatic chamber, the parasitism rate was significantly higher only for galls collected in May (GLM, χ^2^ = 337.23, df = 1, *p* < 0.001), increasing from 31% to 78%, while no significant increase in parasitism rate was detected in June (χ^2^ = 3.09, df = 1, *p* > 0.05) or July (χ^2^ = 3.40, df = 1, *p* > 0.05). Comparing the number of oviposition events by *T. sinensis* and gall toughness, a negative correlation was found (R^2^ = −0.99) (Figure 5).

## 4. Discussion

In this paper, investigations were carried out with ACGW galls of similar size, and only toughness was evaluated for both fresh and dry ACGW galls, providing descriptive information on this gall’s structural trait. As expected, the maximum recorded penetration force (Fmax) increased when testing newly formed ACGW galls from May to December, and showed consistently high results on dry galls. Only the galls collected in November exhibited lower values with respect to previous months (3.52 and 4.48 N); yet, even in that case, the difference between the galls collected in October and November was not statistically significant (*p* > 0.05; Figure 3). In general, when comparing branch and leaf midrib galls, Fmax had similar (*p* > 0.05) values in the first sampling points, except for July (2.25 and 1.79 N, respectively; *p* < 0.001); then, Fmax hardness was significantly higher for branch galls in the three final points (October to December; *p* < 0.05 for all points). Previous investigations by Gil-Tapetado et al. [35] showed an increase in ACGW gall wall resistance to parasitoid attacks over time; however, the authors were not able to find a significant effect of gall toughness on this aspect, probably due to the high variability found with respect to gall thickness or volume. 

*Torymus sinensis* is a parasitoid showing an activity pattern synchronized with gall development [9]. Although no variation in the life cycle of the ACGW has ever been observed, an early emergence of *T. sinensis* (late February-early March) was recently recorded in some Italian chestnut-growing areas when current-year ACGW fresh galls are not available [30]. Currently, such a mismatch has been reported only at a local level, but higher mean temperatures and an increased frequency of climatic extremes are expected to increase synchronization mismatches occurring between tightly interacting species, such as hosts and parasitoids or preys and predators, as in the case of ACGW and its parasitoid. However, the synchronization currently recorded between the ACGW and *T. sinensis* allows the latter to parasitize early in the gall-growing season (April and May) [9]. Our data highlighted that the wall toughness is typically low during these months, leading to a higher success of oviposition by parasitoids during this period. The results obtained when testing toughness in controlled conditions are consistent with the inability of the parasitoid females to oviposit later in the season. The highest percentage of galls were vulnerable in May and June, and no oviposition was recorded in July, highlighting a significant negative relationship between toughness and the number of oviposition events. In all trials carried out in July, the females spent a long time probing and attempting to insert the ovipositor into the galls, and finally rejected them. The number of drills per gall regressed in relation to the toughness, suggesting that galls which are not parasitized at this stage are less likely to be attacked later. Since the galls used in the experiments were of similar size, we assumed that the size did not affect the outcome of the trials.

This is in line with the gall toughness hypothesis, asserting that old galls are not parasitized [36]. Craig et al. [36] determined toughness using an Instron penetrometer and visually observed parasitism rate for the shoot-galling sawfly, *Euura lasiolepis* Smith (Hymenoptera: Tenthredinidae) by *Lathrostizus euurae* (Gravenhorst) (Hymenoptera: Ichneumonidae), reporting that the number of drills per gall regressed according to the toughness, thus suggesting that a low attack rate on large, old galls in the field is probably due to the toughness of these galls. 

The average number of emerging second year *T. sinensis* was in line with previous investigations by Ferracini et al. [22], attesting to 0.4 ± 0.05 adults emerging per 100 cells for winter-collected galls. Excluding first deformation energy (W1), no difference was detected in the wall toughness of galls formed during the previous year when compared to current-year dry galls. This suggests that the mortality of dead newly formed adults inside the galls cannot be explained by the gall tissues being so hard to prevent and counteract *T. sinensis* emergence, negatively affecting diapause. Diapause is a critical state of an insect’s life cycle, when it undergoes the arrestment of growth and/or reproduction to survive adverse environmental conditions and/or food shortage [37]. Although diapause is necessary for surviving the adverse conditions, it is also very costly; extreme temperatures, predation, or depletion of the stored energy reserves of the overwintering individuals can occur [38]. The energy costs during diapause may in fact negatively impact survival, compromising post-diapause development. To our knowledge, from the previous studies conducted to investigate the prolonged diapause for *T. sinensis* [22,23], no other studies have been carried out as of today. Our updated data confirm that the percentage of diapausing larvae has not increased at all over the years, being stable at values of <1%. Thus, the indication given to chestnut growers to leave the resulting material of chestnut pruning in the orchard in order not to compromise the survival of the diapausing individuals may be useful at the beginning of the implementation of biocontrol programs (the dissection of a representative sample of dry galls, e.g., at least 50–100, collected in winter directly in the field can be a useful indicator of the presence of the parasitoid). Conversely, if the biocontrol agent has already been released for at least 3–4 years and is therefore established in a chestnut orchard, the prolonged diapause is negligible and thus not so decisive for the management of ACGW infestations. 

In natural conditions, *T. sinensis* emergence is timed to allow females to parasitize ACGW larvae inside the galls [9]. Thus, the window of vulnerability length may have a deep influence on parasitism rate, highlighting how fresh galls forming in April and May offer few physical barriers in relation to toughness. A similar study design on different *C. sativa* varieties and in other European chestnut growing areas could prove useful for further investigations.

## Data Availability

The data presented in this study are available on request from the corresponding author.
